# N-Acyldopamine induces aggresome formation without proteasome inhibition and enhances protein aggregation via p62/SQSTM1 expression

**DOI:** 10.1038/s41598-018-27872-6

**Published:** 2018-06-25

**Authors:** Gen Matsumoto, Tomonao Inobe, Takanori Amano, Kiyohito Murai, Nobuyuki Nukina, Nozomu Mori

**Affiliations:** 1 Department of Anatomy and Neurobiology, Nagasaki University School of Medicine, 1-12-4 Sakamoto, Nagasaki, 852-8523 Japan; 2 Graduate School of Science and Engineering, University of Toyama, 3190 Gofuku, Toyama-shi, Toyama, 930-8555 Japan; 3 Laboratory of Structural Neuropathology, Doshisha University Graduate School of Brain Science, 1-3 Tatara Miyakodani, Kyotanabe-shi, Kyoto, 610-0394 Japan

## Abstract

Accumulation of ubiquitinated protein aggregates is a common pathology associated with a number of neurodegenerative diseases and selective autophagy plays a critical role in their elimination. Although aging-related decreases in protein degradation properties may enhance protein aggregation, it remains unclear whether proteasome dysfunction is indispensable for ubiquitinated-protein aggregation in neurodegenerative diseases. Here, we show that *N*-oleoyl-dopamine and *N*-arachidonyl-dopamine, which are endogenous brain substances and belong to the *N*-acyldopamine (AcylDA) family, generate cellular inclusions through aggresome formation without proteasome inhibition. Although AcylDA itself does not inhibit proteasome activity *in vitro*, it activates the rearrangement of vimentin distribution to form a vimentin cage surrounding aggresomes and sequesters ubiquitinated proteins in aggresomes. The gene transcription of p62/SQSTM1 was significantly increased by AcylDAs, whereas the transcription of other ubiquitin-dependent autophagy receptors was unaffected. Genetic depletion of p62 resulted in the loss of ubiquitinated-protein sequestration in aggresomes, indicating that p62 is a critical component of aggresomes. Furthermore, AcylDAs accelerate the aggregation of mutant huntingtin exon 1 proteins. These results suggest that aggresome formation does not require proteasome dysfunction and AcylDA-induced aggresome formation may participate in forming cytoplasmic protein inclusions.

## Introduction

Proteostasis alteration is a characteristic of aging brains and causes the deposition of protein inclusions related to various late-onset neurodegenerative diseases^1–3^. Because aging leads to defects in proteostasis resulting in the accumulation of proteotoxic abnormal proteins, it is crucial to understand the molecular mechanisms underlying the imbalance of proteostasis in aging to prevent toxic protein aggregation in neurodegenerative diseases, including Alzheimer’s disease and Huntington’s disease^4^. Neurodegenerative diseases are characterized by neuronal loss or degeneration in a specific region of the brain and most are related to the appearance of ubiquitin-positive inclusions as a remarkable pathology. Protein inclusions in several neurodegenerative diseases, however, appear during middle age, in which ubiquitinated-protein depositions are rarely observed in the healthy brain^5^, indicating that misfolded protein accumulation can be initiated even under normal conditions. The ubiquitin-proteasome system (UPS) directs the primary degradation of misfolded proteins, but this system cannot efficiently degrade aggregated proteins^6–8^. Selective autophagy against protein aggregates (aggrephagy), therefore, compensates for the UPS to degrade aggregated proteins when UPS activity is disrupted, with p62/SQSTM1 playing crucial roles in this compensatory mechanism as a key autophagy receptor in aggrephagy^9–12^. During aggrephagy, p62 requires phosphorylation at S403, which is located in the ubiquitin association domain, to bind to proteasome substrates^13^, which are conjugated with K48-linked polyubiquitin chains. As S403-phosphorylation of p62 stabilizes the interaction between p62 and the polyubiquitin chain, the phosphorylation facilitates autophagosomal engulfment of ubiquitinated-proteins^13,14^. Casein kinase 2 and TANK-binding kinase 1 (TBK1) have been identified as the kinases responsible for S403-phosphorylation in p62^13–16^. Although p62-mediated aggrephagy plays a central role in removing protein aggregates, it remains unclear how misfolded proteins escape aggrephagy and accumulate in cells.

Aggresomes are cellular inducible structures that appear at the juxtanuclear region of the cytoplasm and are defined as “a pericentriolar membrane-free, cytoplasmic inclusion containing misfolded, ubiquitinated protein ensheathed in a cage of intermediate fragments”^17,18^. Aggresome formation occurs when the capacity of the cellular protein degradation systems is exceeded, such as when proteasome activity is inhibited or when aberrant proteins are massively overexpressed. As ubiquitinated-proteins including cytotoxic misfolded proteins are sequestered from the cytoplasmic pool by aggresome formation until the protein degradation systems are restored, it is thought that aggresome formation is a cytoprotective response against proteotoxicity by proteasome dysfunction or the accumulation of polyubiquitinated proteins^19–23^. By recovering protein degradation activity, aggresome structures are dissociated, indicating that aggresome formation is reversible^24^. During aggresome formation, undegraded polyubiquitinated proteins are actively transported to the microtubule organizing center by histone deacetylase 6 (HDAC6) with dynein motor complexes in a microtubule-dependent manner^19,25^. The transported proteins are then confined in a cage of intermediate fragments that is generated by global rearrangement of intermediate filaments, including vimentin, cytokeratin, neurofilament, or glial fibrillary acidic protein (GFAP). Although aggresomes are remarkable cellular structures, the physiological significance and connection with protein degradation deficiency in human diseases remain unknown.

In this study, we found that *N*-oleoyl-dopamine (OLDA) and N-arachidonyl-dopamine (NADA) induce p62/SQSTM1 expression and aggresome formation without proteasome inhibition as their novel functions. These compounds are endogenous lipid derivatives of dopamine belonging to the *N*-acyl-dopamine (AcylDA) family and have been detected in the rodent striatum^26,27^. AcylDAs are known as ligands capable of activating both the cannabinoid type 1 (CB1) receptor and transient receptor potential cation channel subfamily V member 1 (TRPV1) receptor as endocannabinoids/endovanilloids as well as *N*-arachidonyl ethanol amine (AEA)^26,28,29^. Although AcylDA may function as an endocannabinoid, AEA induces neither p62 expression nor aggresome formation. AcylDA itself does not directly inhibit proteasome activity *in vitro*, suggesting that proteasome dysfunction is dispensable for aggresome formation. AcylDA also activates p62 gene expression but not other autophagy receptors, such as OPTN, NBR1, or TAX1BP1. Interestingly, AcylDA-induced p62 proteins are highly S403-phosphorylated in a TBK1-dependent manner and sequester ubiquitinated proteins in aggresomes. Genetic disruption of p62 prevents the sequestration of ubiquitinated-proteins in aggresomes. Super-resolution microscopic analysis revealed that AcylDA-induced aggresomes are composed of clustered p62 bodies in the vimentin cage and aggrephagy occurs outside of the aggresome. Furthermore, AcylDAs enhance the aggregation of polyglutamine expanded huntingtin exon 1 fragments. We suggest that cells can create protein aggregates or inclusions by sequestering ubiquitinated proteins in aggresomes without impairing protein degradation systems and that AcylDA-induced aggresome formation is involved in a pathway that controls protein inclusion formation in neurodegenerative diseases.

## Results

### Screening for p62/SQSTM1 inducer using pharmacologically active compound library

**Figure 1:**
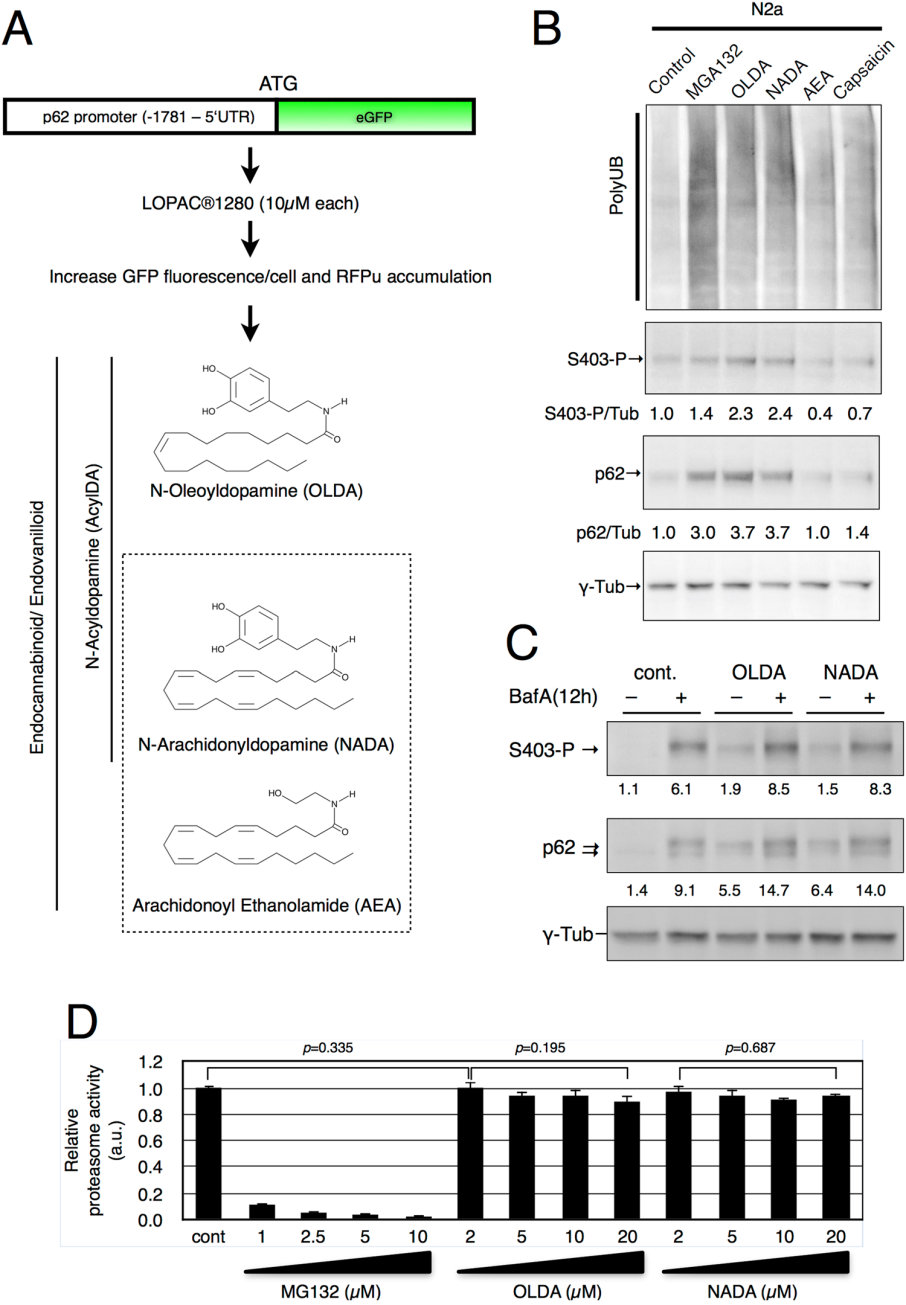
Drag screening for p62 promoter activation compounds. (**A**) A scheme of p62 promoter (−1781 to 5′ untranslated region of p62 exon 1)-driven EGFP gene (p62PR-EGFP) and drug screening using LOPAC1280® library. The compounds that induced GFP expression and RFPu accumulation were selected and the chemical structures of OLDA, NADA, and AEA are shown (**B**). Normal N2a cells were treated with 5 µM OLDA, 5 µM NADA, 10 µM AEA, 50 µM capsaicin, or 1 µM MG132 for 12 h as indicated (**C**). N2a cells were treated with 5 µM OLDA or 5 µM NADA and with or without 1 µM bafilomycinA1 (BafA) simultaneously for 12 h. S403-phosphorylated p62 (S403-P) was detected with monoclonal anti-phospho-p62 (S403) antibody (4F6). Total p62 was visualized with anti-p62 (polyclonal). The ratio between relative amounts of phosphorylated or total p62 normalized to γ-tubulin is shown (**B,C**). (**D**) *In vitro* proteasome activity assay was carried out using purified yeast 26S proteasomes with 2 nM proteasome, 125 µM Suc-LLVY-AMC, and compounds as indicated incubated at 30 °C for 30 min followed by calculation of the relative proteasome activity. Data are shown as the mean ± SEM values of 9–12 measurements under each condition and p values (Student’s *t*-test) are shown.

To identify chemical compounds that induced p62 expression, we first generated a p62 expression reporter construct (p62PR-EGFP), in which the human p62 promoter^30^ drove enhanced green fluorescent protein (EGFP) expression as shown in Fig. 1A, and the reporter construct was stably transfected into Neuro2a (N2a) cells. Because proteasome inhibition activates p62 expression^13^, we first tested whether p62PR-EGFP cells responded to protein degradation deficiency. Upon MG132 treatment, GFP fluorescence in p62PR-EGFP cells was increased in response to EGFP protein expression (Supplementary Fig. S1). By measuring GFP fluorescence in each p62PR-EGFP cell, we performed chemical compound screening using the LOPAC1280 library which contains 1280 pharmacologically active compounds (Fig. 1A). We found 78 compounds that significantly induced or reduced GFP fluorescence in the cells. To determine whether these candidate compounds modified ubiquitinated-protein degradation, we constructed an N2a-derived UPS reporter cell line in which a short degron, CL1, conjugated to red fluorescent protein (RFPu) was stably transfected, and measured RFPu fluorescence after 24-h treatments with the candidate compounds. Because RFPu was unstable, like GFPu^31^, and degraded by proteasomes following polyubiquitination, MG132 treatment prevented RFPu degradation and intensified RFPu fluorescence signals at the perinuclear region of the cell (Supplementary Fig. S1). Through compound screening with the RFPu cell line, we found that OLDA significantly increased RFPu fluorescence in the perinuclear region (Supplementary Fig. S1), while other library chemicals did not apparently alter RFPu stability. As RFPu accumulation is related to proteasome dysfunction and p62 plays a key role in compensating for the UPS under stress conditions, OLDA may regulate protein degradation through the p62-mediated selective autophagy pathway. Therefore, we focused on this compound for further analysis.

### N-Acyldopamines (AcylDAs) induce p62 not as endocannabinoids nor endovanilloids

To investigate whether OLDA controls p62-mediated selective autophagy, we measured the S403-phosphorylated p62 protein level using an autophagy-deficient cell line (N2a-miR-Atg5) in which Atg5 was stably knocked down^13^, as S403-phosphorylated p62 proteins were dominantly incorporated into autophagosomes^14^. OLDA treatment increased the amount of both total and S403-phosphorylated p62 proteins and enhanced S403-phosphorylation of p62 in a dose-dependent manner (Supplementary Fig. S1)

NADA is also known as an endogenous brain substance belonging to the AcylDA family^26,28^ and both OLDA and NADA are ligands for CB1 cannabinoid receptors and TRPV1 vanilloid receptors, although their physiological significance remains unclear^27^. To determine if OLDA induces p62 expression through cannabinoid- or vanilloid receptor-mediated signaling pathways, we measured the accumulation of total and S403-phosphorylated p62 proteins by treatment with *N*-arachidonyl-ethanolamine (anandamide; AEA) or capsaicin as a control for the CB1 or TRPV1 agonist, respectively (Fig. 1B). Both OLDA and NADA treatment induced accumulation of total and S403 phosphorylated p62 proteins. However, AEA and capsaicin did not alter p62 levels. These results suggest that NADA has the same p62 induction effects as OLDA, but the function is neither mediated by the cannabinoid nor the vanilloid signaling pathways. Interestingly, AcylDA-treated cells did not accumulate polyubiquitinated proteins to the same extent as MG132-treated cells, but significant amounts of ubiquitinated proteins remained in AcylDA-treated cells.

### AcylDA does not inhibit constitutive autophagy or proteasome activity

As S403-phosphorylated p62 is dominantly engulfed by autophagosomes^13^ and p62 proteins are constitutively degraded through constitutive autophagy under normal conditions^10,32^, the accumulation of S403-phospho-p62 proteins under AcylDA-treated conditions may be a consequence of autophagy inhibition. To determine whether AcylDAs interfere with autophagy flux, we treated N2a cells with both AcylDA and Bafilomycin A1 (BafA), an inhibiter of autophagosome-lysosome fusion, and evaluated the accumulation of S403-phosphorylated p62 by BafA treatments. S403-phosphorylated p62 degradation was blocked by BafA, suggesting that p62 was degraded through autophagy during AcylDA treatment and that AcylDAs did not inhibit constitutive autophagy (Fig. 1C).

Because proteasome inhibition also induces p62 and its S403-phosphorylation, AcylDAs may inhibit proteasome activity. To determine whether AcylDA directly inhibits proteasome activity, we performed an *in vitro* proteasome activity assay using purified yeast proteasomes. As shown in Fig. 1D, AcylDAs did not inhibit proteasome activity even when a 4-fold higher concentration was used in the *in vitro* assay compared to in the cell culture experiments, whereas 1 µM MG132 treatment, the concentration was used to treat N2a cells, inhibited nearly 90% of proteasome activity *in vitro*. These results demonstrate that AcylDA does not directly inhibit proteasome activity. We also confirmed in cells that polyubiquitinated proteins accumulation was much lower under AcylDA-treated conditions than under MG132 treatment (Fig. 1B and Supplemental Fig. S1). These data indicate that AcylDA-mediated p62 induction is not related to the deficiency of ubiquitinated-protein degradation.

### AcylDA induces aggresome formation with S403-phospho-p62 accumulation

**Figure 2:**
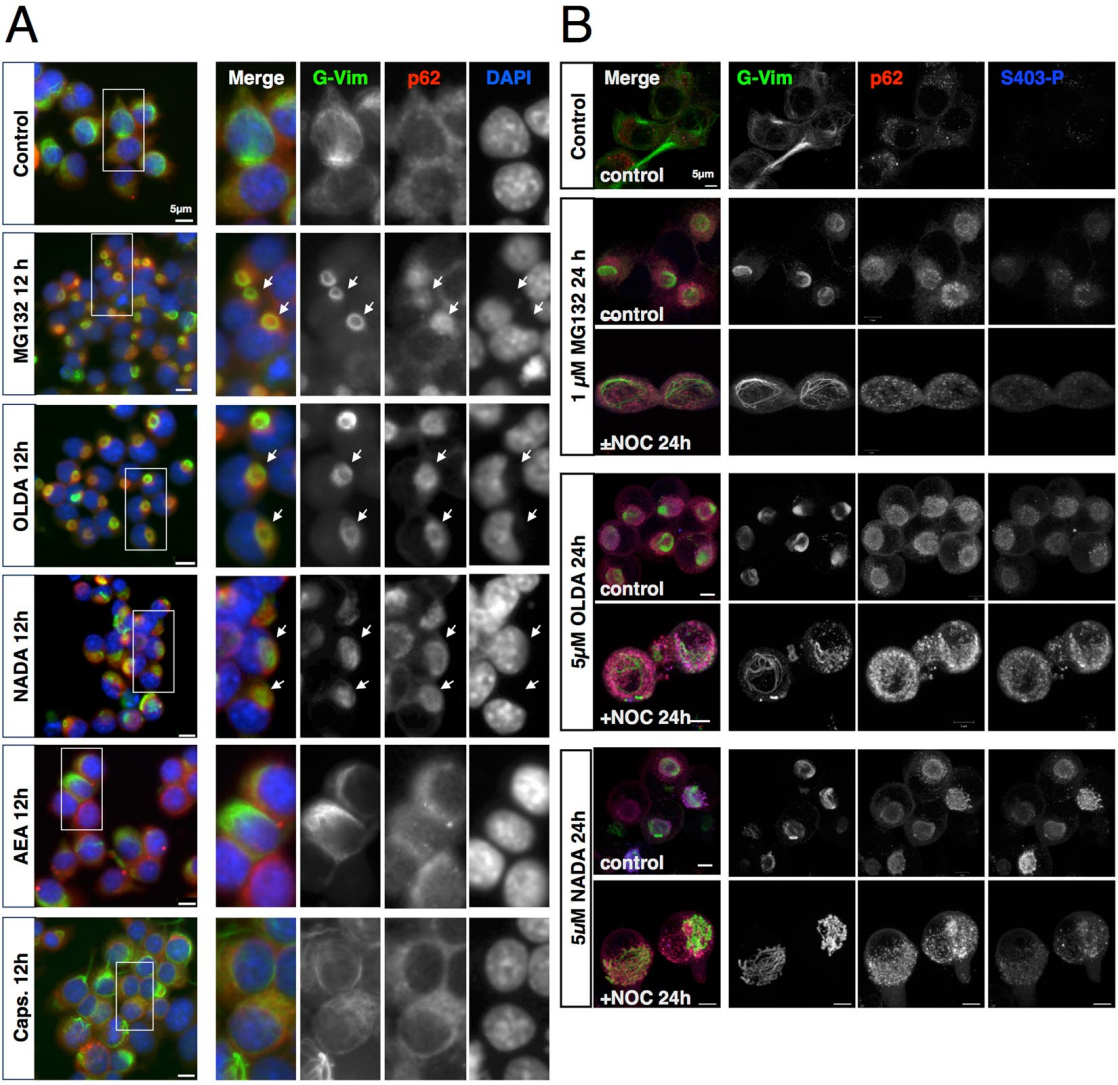
AcylDAs induce aggresome formation without proteasome inhibition. (**A**) N2a cells stably expressing GFP-vimentin were treated with 5 µM OLDA, 5 µM NADA, 10 µM anandamide (AEA), 50 µM capsaicin, or 1 µM MG132 for 12 h as indicated. G-vimentin (G-Vim, green in merged images), p62 (red), and DAPI (blue; nucleus) were visualized by fluorescence microscopy. The arrows indicate aggresomes surrounded by the vimentin cage. (**B**) GFP-vimentin cell lines were treated with 1 µM MG132, 5 µM OLDA, or 5 µM NADA and with or without 10 µM nocodazole (+NOC) simultaneously for 24 h as indicated. G-Vimentin (G-Vim, green), p62 (red), and S403-phospho-p62 (S403-P; blue) were visualized by confocal microscopy. Scale bars represent 5 µm.

Through screening, we observed cytoplasmic accumulation of RFPu following OLDA treatment (Supplemental Fig. S1). This indicates that AcylDAs play a role in the sequestration of damaged proteins into aggresomes by inducing p62 expression. The aggresome is an inducible cellular structure that differs from cellular puncta containing protein aggregates and is characterized by a vimentin cage structure. To determine whether AcylDA induces aggresome formation, we monitored vimentin cage formation using a N2a cell line stably expressing GFP-vimentin (G-vimentin) (Fig. 2A). Under normal conditions, G-vimentin formed filamentous structures and distributed throughout the cytoplasm as intermediate filaments. Proteasome inhibition by MG132 treatment resulted in the apparent reorganization of G-vimentin, which formed a sphere cage structure as the remarkable feature of aggresomes. Both OLDA and NADA induced vimentin cage formation, suggesting that AcylDA treatment stimulated aggresome formation, although AcylDAs did not inhibit proteasome activity. In the AcylDA-induced aggresomes, p62 proteins were concentrated inside of the vimentin cage as well as MG132-induced aggresomes. To demonstrate that the AcylDA-induced vimentin cage represents the aggresome, we examined the localization of centrosomes (γ-tubulin) and HDAC6 as aggresome markers and confirmed that the centrosomes and HDAC6 were detected inside of the vimentin cage (Supplemental Fig. S2). As expected, AEA and capsaicin did not alter the vimentin distribution, indicating that CB1- and TRPV1-mediated signaling is not involved in the aggresome formation. Because cells overexpressing p62 proteins do not contain aggresomes as described previously^9,13,33^, p62 induction itself does not cause AcylDA-induced aggresome formation.

As aggresome formation is driven by microtubule-dependent retrograde transportation^17,19^, we investigated whether AcylDA-induced aggresome formation depends on the microtubules (Fig. 2B). Disruption of microtubules by nocodazole treatment interfered with AcylDA-induced G-vimentin reorganization and p62 concentration at the perinuclear region, suggesting that both vimentin cage formation and p62 accumulation required microtubule-dependent active transportation during AcylDA-mediated aggresome formation.

### p62 is essential for the accumulation of ubiquitinated proteins in aggresomes

**Figure 3:**
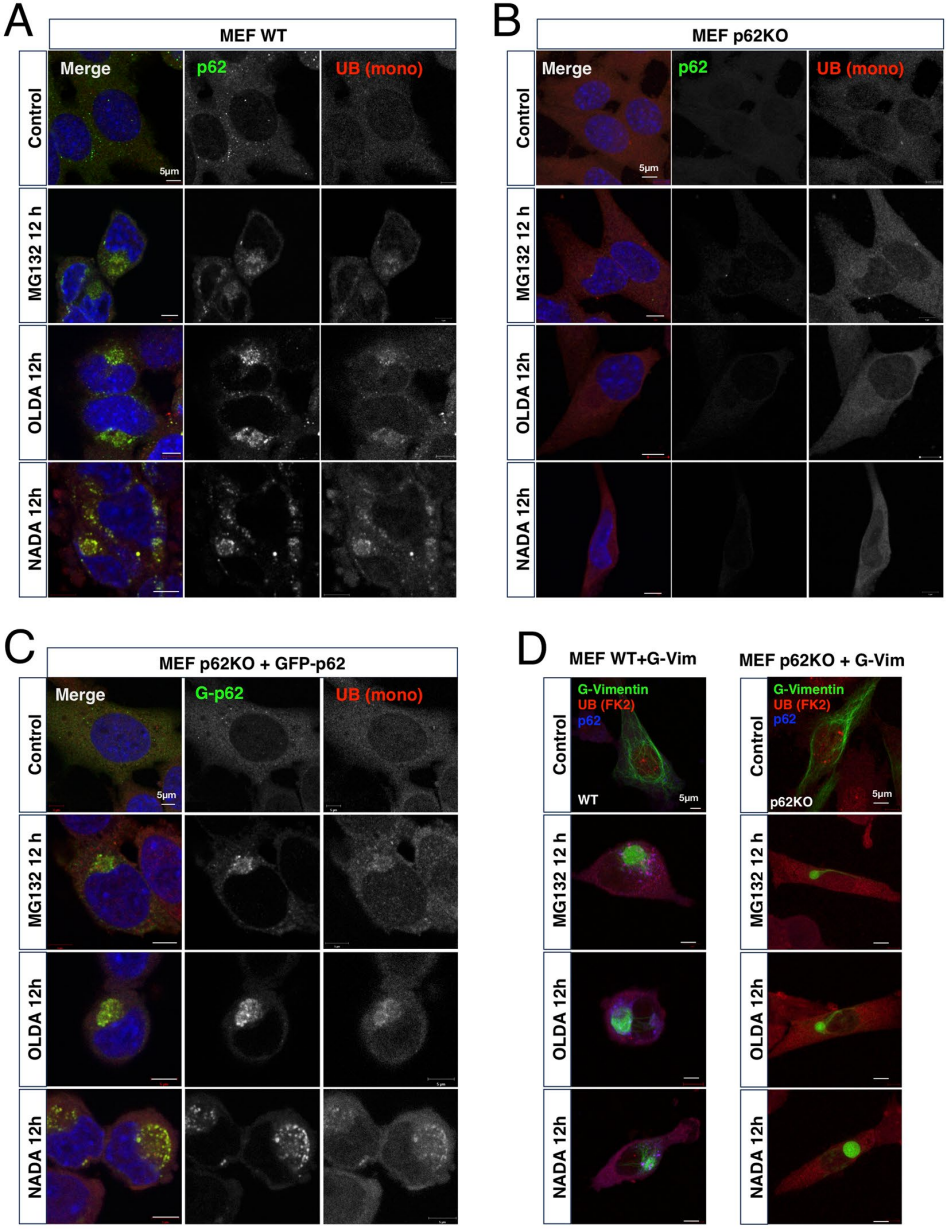
p62/SQSTM1 is essential for ubiquitinated protein accumulation in aggresomes, but is not required for vimentin cage formation. Wild-type MEF cells (**A**), p62 knockout MEF cells (**B**), or p62KO + GFP-p62 MEF cells (**C**) were treated with 1 µM MG132, 5 µM OLDA, or 5 µM NADA for 12 h as indicated. Total p62 (green), ubiquitin monomer (UB (mono), red), and DAPI (nucleus, blue) were visualized by confocal microscopy as indicated. (**D**) GFP-Vimentin plasmid was transiently transfected in wild-type MEF or p62KO MEF cells for 24 h and treated with 1 µM MG132, 5 µM OLDA, or 5 µM NADA for 12 h as indicated. GFP-Vimentin (green), multi-ubiquitin (UB (FK2), red), and p62 (blue) were visualized by confocal microscopy and merged images are shown as indicated. Scale bars represent 5 µm.

To investigate the relationship between AcylDA-meditated p62 induction and aggresome formation, we monitored aggresome formation in p62 KO MEF cells. In wild-type MEF cells, aggresome formation was induced by AcylDAs and ubiquitinated proteins accumulated in the aggresome (Fig. 3A). However, ubiquitinated-protein accumulation in both MG132- and AcylDA-induced aggresomes was completely disrupted in p62 KO MEF cells (Fig. 3B), indicating that p62 plays an essential role in the sequestration of ubiquitinated proteins in aggresomes. The requirement for p62 proteins in the sequestration of ubiquitinated proteins in aggresomes was confirmed by GFP-p62 stably expressing p62 knockout (KO) mouse embryonic fibroblast (MEF) cells. In this cell line, as GFP-p62 is constitutively expressed under the cytomegalovirus (CMV) promoter, which is not regulated by AcylDAs, p62 protein production was not altered by AcylDA treatments. The stable expression GFP-p62 suppressed the loss of ubiquitinated-protein accumulation during aggresome formation in p62 KO MEF cells (Fig. 3C). To investigate whether vimentin cage formation occurs because of p62 protein and ubiquitinated-protein accumulation, we transiently transfected G-vimentin into wild-type or p62KO MEF cells and monitored the vimentin distribution under AcylDA- or MG132-treated conditions. G-Vimentin changed its distribution drastically and formed a cage structure at the perinuclear region, even in p62 KO MEF cells, following AcylDA or MG132 treatment (Fig. 3D). These results suggest that p62 is not required for vimentin reorganization in aggresome formation.

### AcylDA-induced aggresome has distinct propensities from MG132-induced aggresome

**Figure 4:**
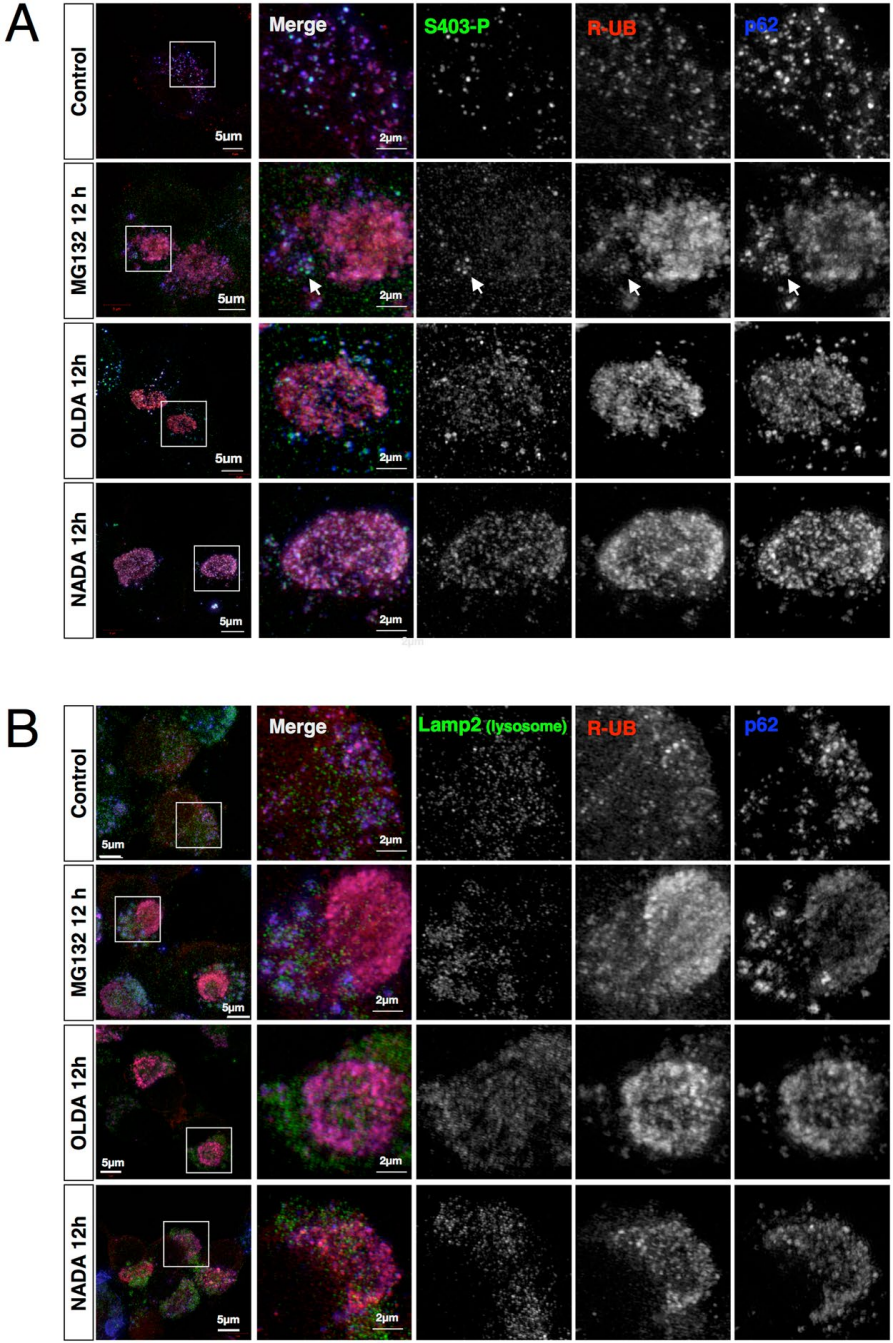
Super-resolution microscopy (SR-SIM) revealed that the aggresome consists of p62 bodies containing p62 and ubiquitinated proteins. N2a cells stably expressing RFP-fused ubiquitin (R-UB) were treated with 1 µM MG132, 5 µM OLDA, or 5 µM NADA for 12 h as indicated. S403-phosphorylated p62 (S403-P; green) in (**A**), Lamp2A (lysosome; green) in (**B**), RFP-UB (red), and total p62 (blue) in (**A,B**) were visualized by super-resolution structured illumination microscopy (SR-SIM) as indicated. Magnified images in square box (10 µm each side) on left panels are shown. Scale bars represent 5 µm in wide images or 2 µm in magnified images as indicated.

The aggresome has been interpreted as a cell-protective response against proteotoxicity caused by the accumulation of misfolded proteins when the cellular capacity of the protein degradation system is exceeded. Therefore, induction of aggresome formation may require the dysfunction or saturation of proteasomes. However, our results suggest that proteasome dysfunction is not essential for aggresome formation. To dissect the structural characteristics of AcylDA-induced aggresomes in cells, we performed high-resolution analysis using super-resolution structured illumination microscopy (SR-SIM) to examine a N2a cell line stably expressing RFP-ubiquitin (R-UB) (Fig. 4). In the MG132-induced aggresome, R-UB was completely co-localized with p62, while S403-phosphorylated p62 was rarely detected in the aggresome. However, S403-phosphorylated p62 proteins co-localizing with R-UB were detected in p62 bodies localized outside the MG132-induced aggresome (Fig. 4A). In contrast, OLDA- or NADA-induced aggresomes consisted of p62-positive puncta containing both R-UB and S403-phosphorylated p62 (Fig. 4A,B).

To investigate whether ubiquitinated proteins in the AcylDA-induced aggresome can be degraded through autophagy-lysosome pathway, we evaluated lysosome localization by SR-SIM (Fig. 4B). In MG132-treated cells, lysosomes surrounded the p62 bodies outside the aggresome. This observation supports that ubiquitinated proteins sequestered in aggresomes are eventually degraded by aggrephagy. AcylDA-induced aggresomes also showed a similar propensity of MG132-induced aggresomes, suggesting that ubiquitinated proteins were degraded through p62-mediated aggrephagy in the cytoplasm during aggresome formation. Notably, cytoplasmic p62 bodies were co-localized with GFP-LC3 in OLDA-treated GFP-LC3 stably expressing N2a cells (Supplemental Fig. S3), indicating that p62 bodies were surrounded by autophagosomes in the cytoplasm and then fused with the lysosome.

### S403-phosphorylation of p62 is dispensable for AcylDA-induced aggresome formation

To determine if S403-phosphorylation is critical for AcylDA-induced aggresome formation, we inhibited S403-phosphorylation by adding BX795, a TBK1 inhibitor, during AcylDA treatments and monitored aggresome formation (Supplementary Fig. S4). Aggresome formation did occur even under BX795-treated conditions, although p62 phosphorylation at S403 was prevented. These results indicate that S403-phosphorylation of p62 occurs independently of aggresome formation and TBK1 directs S403-phosphorylation during AcylDA-dependent aggresome formation.

### AcylDA activates p62 transcription, but not other autophagy receptors

**Figure 5:**
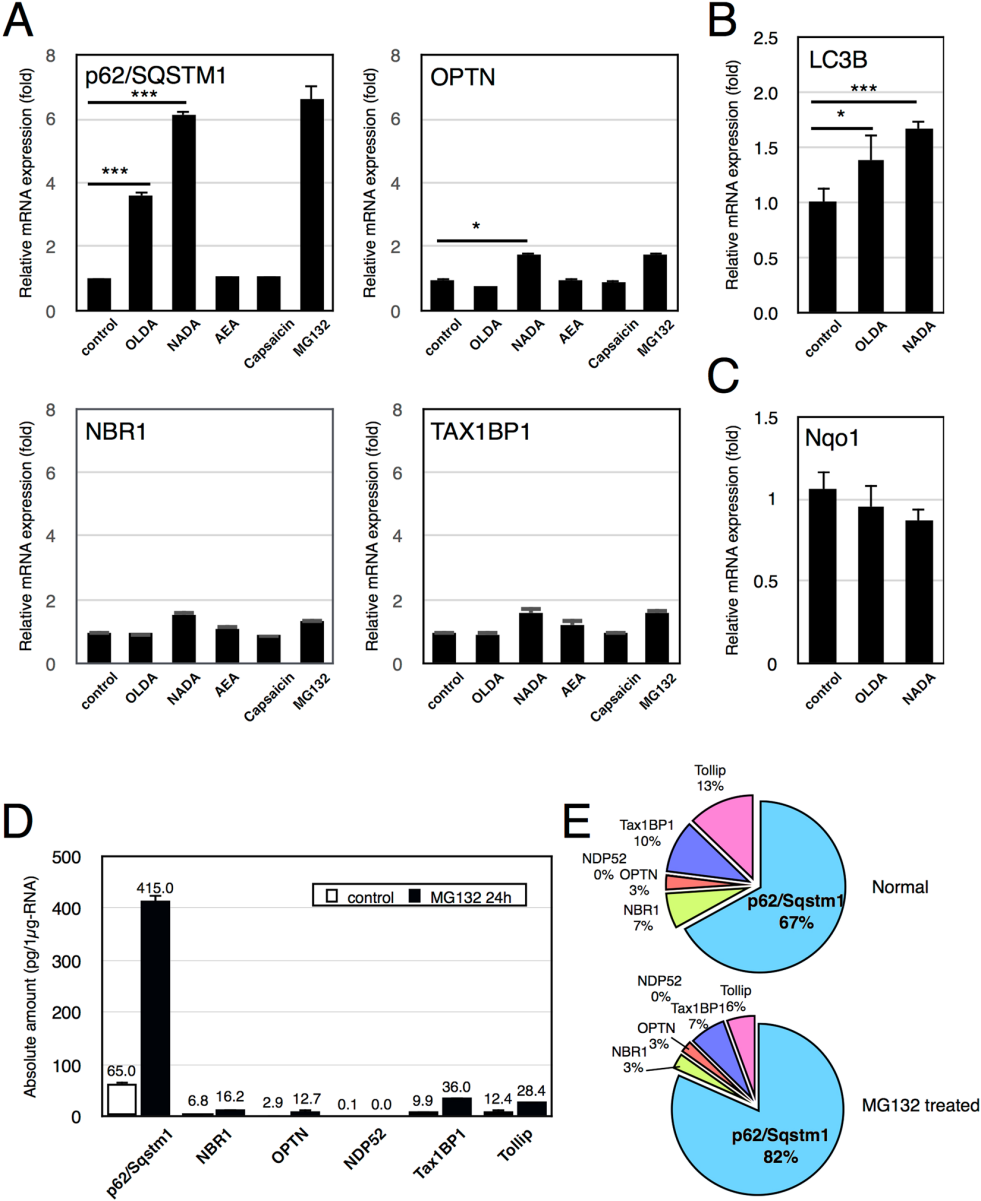
AcylDA induce p62 expression in transcription level but does not affect the expression of other autophagy receptors. (**A**) Relative mRNA levels of autophagy receptors in N2a cells treated with 5 µM OLDA, 5 µM NADA, 10 µM anandamide (AEA), 50 µM capsaicin (Caps), or 1 µM MG132 for 12 h as indicated were measured by quantitative RT-PCR (qPCR) analysis. mRNA amounts of each autophagy receptor were normalized to Gapdh. (**B**) Relative mRNA level of LC3B in N2a cells treated with 5 µM OLDA or 5 µM NADA for 24 h was measured by qPCR and normalized to Gapdh. (**C**) Relative mRNA levels of Nqo1 in N2a cells treated with 5 µM OLDA or 5 µM NADA for 12 h were measured by quantitative RT-PCR and the amount was normalized to Gapdh. Error bars represent SEM (n = 4), and p-value (Student’s *t*-test) is shown. *and ***represent p < 0.05 and p < 0.01 (**D**). Total RNAs were prepared from N2a cells treated with or without 1 µM MG132 for 24 h. The absolute amounts of mRNA of autophagy receptors in 1 µg-total RNA of N2a cells were calculated by quantitative RT-PCR analysis. GFP-fused autophagy receptor plasmids were used as quantification standards and each plasmid amount was normalized to the copy number of the GFP gene. Gapdh mRNA was 2.05 ± 0.78 ng/µg-RNA of N2a cells. The mRNA amounts (pg/µg-total RNA) of each autophagy receptor are shown. (**E**) Ratio of mRNA content of ubiquitin-dependent autophagy adapters in N2a.

AcylDA treatment increases the amount of S403-phospho-p62 that is utilized as an autophagy receptor during selective autophagy. To test whether AcylDA-dependent transcriptional activation affects the expression of other autophagy receptors, the levels of NBR1, OPTN, and TAX1BP1 mRNA were measured by quantitative reverse transcription (RT)-polymerase chain reaction (PCR) (Fig. 5A). The transcription of p62 was clearly activated by the OLDA, NADA, and MG132 treatments. Compared to p62/SQSTM1, the expression levels of other autophagy receptors were slightly increased by AcylDA treatments, but the fold-change was less than 2, even under proteasome inhibition conditions. The AEA and capsaicin treatments did not affect the transcription of all autophagy receptors, as expected. These results suggest that AcylDAs specifically induce p62 expression, but not the expression of other autophagy receptors.

Because autophagic degradation occurred after aggresome formation as shown in Fig. 4B, we examined LC3B expression in AcylDA-treated cells. The LC3B mRNA level significantly increased during 24-h AcylDA treatments (Fig. 5B), although the induction level was less than 2-fold.

Nrf2 is known to regulate the p62 promoter. To determine if Nrf2 is related to AcylDA mediated p62 expression, the expression of Nqo1, which is also regulated by Nrf2, was measured (Fig. 5C). The Nqo1 mRNA content was not altered by AcylDA treatment, suggesting that Nrf2 stabilization is not responsible for AcylDA-mediated p62 induction.

### p62/SQSTM1 is the most abundant autophagy receptor in N2a cells

Under proteasome-inhibited conditions, autophagy receptors, particularly p62 and NBR1, play critical roles in compensating for the UPS and are degraded together with ubiquitinated-proteins by aggrephagy^9,10,13,34,35^. As autophagy receptors are not recycled during selective autophagy, the gene expression of autophagy receptors is essential for inducing selective autophagy processes and the contribution of each autophagy receptor in aggrephagy can be evaluated by its gene expression level. We measured the absolute amount of mRNA of all known ubiquitin-dependent autophagy receptors in N2a cells by quantitative RT-PCR (Fig. 5D,E). Under normal conditions, the p62 mRNA level was 65.05 ± 3.38 pg/µg-RNA, accounting for 67% of the six ubiquitin-dependent autophagy receptors. NDP52 (Calcoco2) mRNA was nearly undetectable (0.06 ± 0.05 pg/µg-RNA) in N2a cells. Because N2a cells are of neuronal origin, this result is consistent with those of previous reports^36^ indicating that NDP52 is not required for selective autophagy in neuronal cells. Following MG132 treatment, p62 mRNA was obviously induced (415.0 ± 9.52 pg/µg-RNA; up to 82% of the total mRNA amount of six receptors) and p62 protein was accumulated, whereas only minor mRNA induction was observed for other autophagy receptors. As autophagy receptors are degraded together with autophagic substrates during selective autophagy, these results suggest that p62 is the most abundant autophagy receptor and is primarily utilized for autophagic degradation of ubiquitinated-proteins under proteotoxic stress conditions.

### AcylDA enhances aggregation of mutant huntingtin

**Figure 6:**
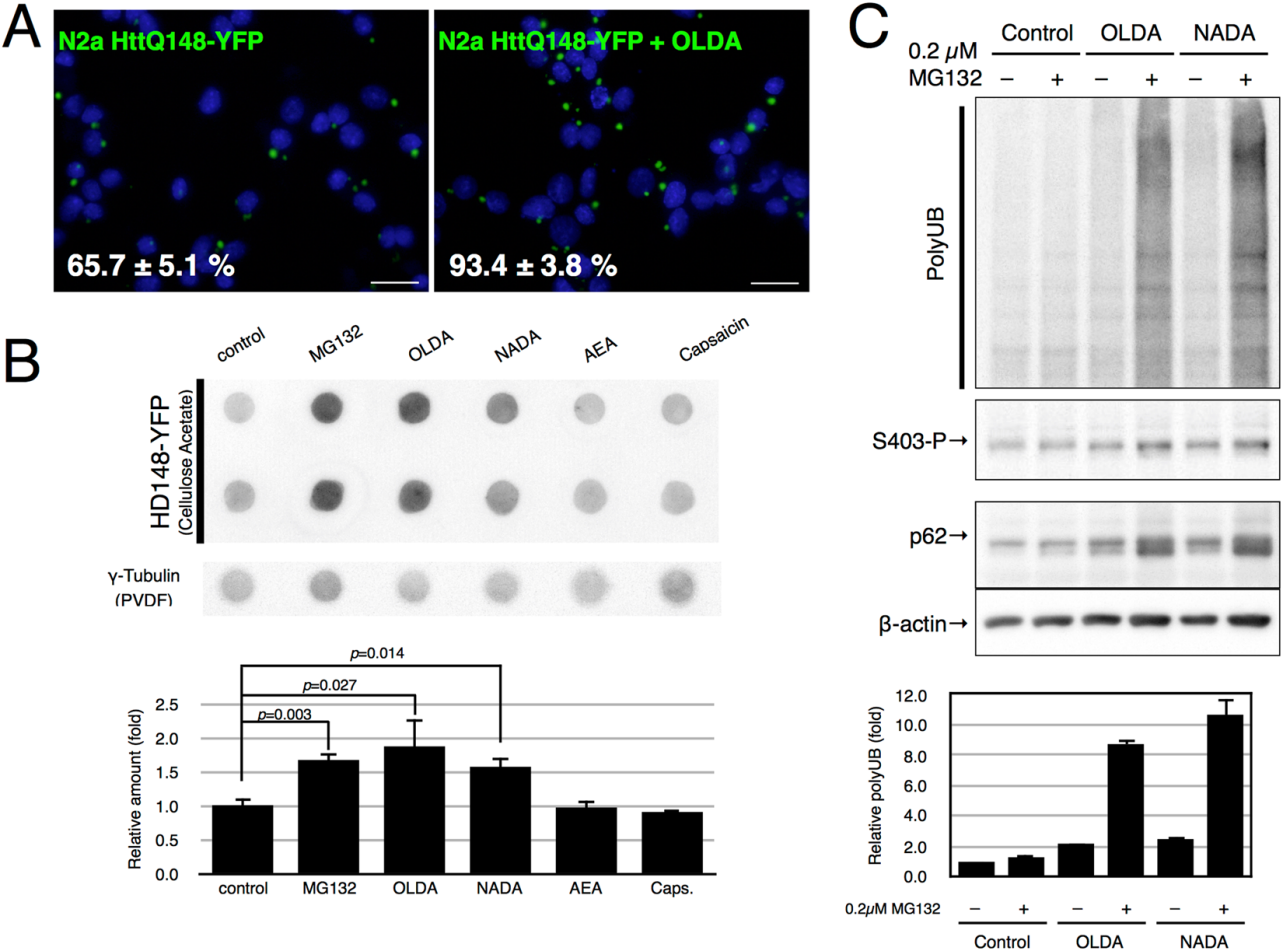
AcylDA accelerates huntingtin aggregation. (**A**) N2a cells stably expressing huntingtin-ex1-Q148-YFP (N2a HttQ148-YFP) were treated with 3 µM OLDA for 3 days after Htt-YFP induction by doxycycline. Cells with Htt aggregates were counted (n > 100) and the number of aggregates was divided by the number of nuclei. (**B**) N2a HttQ148-YFP cells were treated with 5 µM OLDA, 5 µM NADA, 10 µM AEA, 50 µM capsaicin, or 1 µM MG132 for 48 h as indicated. Htt aggregates were visualized with anti-GFP antibody by filter trap assay. As a sample control, dot blot of the same sample on the polyvinylidene fluoride membrane is shown. The relative amount of HttQ148-YFP is shown. Error bars represent SEM (n = 4) and p values (Student’s *t-*test) are shown. (**C**) N2a cells were treated with 0.2 µM MG132 and/or 5 µM OLDA or 5 µM NADA for 12 h as indicated. Total ubiquitinated proteins (polyUb; monomer), S403-phospho-p62 (S403-P), total p62, and β-actin are shown as indicated. Relative amounts of polyubiquitinated proteins (polyUb) in duplicated analysis are depicted. Error bars represent SD (n = 2).

As p62 plays critical roles in aggrephagy, its loss enhances protein aggregation of aggregation-prone proteins as previously reported^37,38^. Induction of p62 proteins by AcylDAs may prevent polyglutamine aggregation by accelerating their degradation. To test whether AcylDA treatment suppresses polyglutamine aggregation, we evaluated the aggregation efficiency of the yellow fluorescent protein (YFP)-fused mutant huntingtin exon1 fragment with 148 polyglutamine repeats (HttQ148-YFP) using a doxycycline (Dox)-inducible N2a cell line expressing HttQ148-YFP^13^. Unexpectedly, OLDA treatment together with Dox treatment obviously induced HttQ148-YFP protein aggregation. More than 90% of the cells formed Htt aggregates by OLDA treatments based on our fluorescence microscopy results after 3 days of induction with Dox, whereas approximately 65% of HttQ148-YFP cells showed aggregates without OLDA (Fig. 6A). To confirm these observations, we performed a filter trap assay to quantify the aggregation efficiency and found that AcylDA significantly induced Htt aggregation to a similar level as proteasome inhibition (Fig. 6B). As AEA or capsaicin treatment rarely altered Htt aggregation, the CB1 and TRPV1 signaling pathways are not related to aggregation processes. These results indicate that AcylDA does not prevent polyglutamine aggregation, but rather accelerates aggregation processes.

As S403-phosphorylated p62 proteins efficiently bind to K48-linked polyubiquitin chains conjugated to proteasome substrates, AcylDA-induced S403-phosphorylated p62 proteins may deprive the substrates of proteasomes and isolate them in aggresomes. If so, excess S403-phosphorylated p62 proteins disturb the efficient degradation of ubiquitinated-proteins by proteasomes. To test this possibility, we monitored the accumulation of ubiquitinated-proteins in cells with weak proteasome activities, which were treated with a low concentration of MG132 to partially inhibit proteasome activity (Fig. 6C). In the cells treated with 0.2 µM MG132, ubiquitinated-proteins were efficiently degraded, indicating that N2a cells maintained a sufficient capacity for proteasomal degradation activities. However, the accumulation of ubiquitinated proteins in cells treated with both AcylDA and low concentration of MG132 clearly increased, whereas OLDA or NADA treatment showed similar levels of accumulation in control cells. These results suggest that AcylDA-induced p62 proteins sequester ubiquitinated proteins in aggresomes before their proteasomal degradation, although the proteasomes in the cells could degrade these proteins.

## Discussion

In this study, we demonstrated that AcylDAs, such as OLDA and NADA, induce aggresome formation and that p62/SQSTM1 plays critical roles in ubiquitinated-protein sequestration in aggresomes. Aggresomes are defined by two major components: ubiquitinated-protein accumulation and a cage structure of intermediate filaments. As the loss of p62 results in the disappearance of ubiquitinated-protein accumulation in aggresomes but does not influence vimentin cage formation (Fig. 3), p62 is an essential component of aggresomes to sequester ubiquitinated-proteins, but does not regulate aggresome formation. These findings are consistent with the previous observations that the simple overexpression of p62 or p62 accumulation by autophagy disruption does not induce aggresome formation^10,13,39^. Although aggresome formation is thought to cause ubiquitinated-protein accumulation, our results revealed that ubiquitinated-protein accumulation is dispensable for aggresome formation and cage formation with intermediate filaments.

Because both p62 and intermediate filaments are commonly found in intracellular inclusion bodies in neurodegenerative diseases^40,41^, aggresome formation may be related to the creation of disease-associated cytoplasmic inclusion bodies including Lewy bodies in Parkinson’s disease and dementia with Lewy bodies. As Lewy bodies appear in dopaminergic neurons in Parkinson’s disease, the AcylDA production level may be associated with Lewy body generation and disease progression if AcylDA production is regulated and increased in aged brains. Further studies are needed to clarify whether AcylDA-mediated aggresome formation and p62 expression occur in human brains.

NADA and OLDA are present in mammalian brain tissues, specifically in the striatum, and are known as endocannabinoids and endovanilloids^26–29^. Our results present additional functions for these compounds as aggresome formation inducers which does not occur through cannabinoid- nor vanilloid-receptor mediated signaling pathways. It has been proposed that NADA is generated in the brain by conjugating arachidonic acid to dopamine and that fatty acid amino hydroxylase (FAAH) catalyzes this conjugation, rather than conversion from acyl-tyrosine by tyrosine hydroxylase^28,42^. This hypothesis is supported by the a study showing that FAAH KO results in the loss of NADA in the striatum, whereas AEA is increased in the striatum of FAAH KO mice^43^. As the FAAH-mediated hydrolysis of AEA is a source for NADA production^42^, the amount of NADA may reflect the local concentration of AEA and dopamine. Modulation of dopaminergic neuron activities by the endocannabinoid system is thought to be involved in several behavioral functions^44^. As AEA administration increases neurotransmission in the mesolimbic dopamine reward system in the nucleus accumbens shell through the CB1 receptor-mediated pathway^45^, it is possible that the local concentration of NADA is transiently increased by FAAH-mediated NADA production. If AcylDA-mediated aggresome formation plays a role in inclusion formation in aged brains, FAAH inhibition may be beneficial for reducing inclusion formation as a therapy for this condition.

Because protein inclusions are remarkable hallmarks of many neurodegenerative diseases, it is thought that aging decreases the cellular protein degradation properties and that impaired protein degradation systems induce protein aggregation. However, the accumulation of ubiquitinated proteins is rarely observed in healthy aged brains and non-affected regions of the brains of patients^5^, indicating that the capacity for protein degradation is not exceeded even in aged brain. This raises the question of how ubiquitinated aggregation-prone proteins escape from degradation systems followed by inclusion body formation in disease-affected neurons. Our results show that NADA and OLDA induce p62 expression and its S403-phosphorylation and accelerate the polyglutamine aggregation processes. AcylDA-induced S403-phosphorylated p62 proteins can capture ubiquitinated proteins and interfere their proteasomal degradation under weak proteasome conditions. These results indicate that AcylDA-induced S403-phosphorylated p62 proteins transiently protect ubiquitinated aggregation-prone proteins from proteasomal degradation by sequestering them in aggresomes. During the sequestration periods, aggregation-prone proteins, e.g. polyglutamine proteins, may begin self-perpetuating aggregation in aggresomes. As aggregation core formation is the rate-limiting step of inclusion formation^46,47^, the transient sequestration and concentration of aggregation-prone proteins may initiate aggregation core formation. Although aggresome formation has cytoprotective roles, it also promotes protein inclusion formation if the cells contain aggregation-prone misfolded proteins. Further studies are needed to elucidate the underlying regulation mechanisms of aggresome formation using AcylDA for understanding the molecular mechanisms and importance of this regulation.

## Material and Methods

### Cell culture and plasmids

All cell lines were maintained at 37 °C in a 5% CO_2_-humidified atmosphere in Dulbecco’s modified Eagle’s medium (Sigma-Aldrich, St. Louis, MO, USA) supplemented with 10% fetal bovine serum (FBS; Gibco, Grand Island, NY, USA), 100 U/mL penicillin, and 100 μg/mL streptomycin (Wako, Osaka, Japan). N2a-derived stable cell lines were maintained by further addition of 200 μg/mL hygromycin and/or 400 μg/mL G418 (Wako). The human p62 promoter (p62PR; −1781 to +46 region of upstream of p62 gene)^30^ was cloned by PCR using the genomic DNA of HeLa cells. The p62PR was replaced with the CMV promoter of pEGFP-N1. The p62PR-EGFP integrated N2a cell line was prepared by transfection with pp62PR-EGFP plasmid into N2a cells followed by drug screening with 400 μg/mL G418 and single colonies expressing GFP were subcloned twice. The mRFP-miR-Atg5 or JTI-G-LC3 stably expressing N2a cell line was previously described^13^. GFP-vimentin or R-UB stably expressing N2a cell lines were generated by transfection with pGFP-mVimentin or pmRFP-ubiquitin-wt plasmid, respectively, into N2a cells followed by drug selection with 600 μg/mL G418 and single colonies were subcloned twice. MEF cells, both wild-type and p62 KO, were described previously^14^ and p62KO + G-p62 MEF cells were generated by stable transfection with JTI-CMV-G-p62 plasmids using the Jump-in system (Thermo Fisher Scientific, Waltham, MA, USA). Plasmid transfection was carried out using lipofectamine 2000 or 3000 reagent (Thermo Fisher Scientific) for N2a cells or by electroporation (NEPA-GENE, Chiba, Japan) for MEF cells.

For cloning of autophagy receptors, each gene was amplified using specific primers including the first ATG to the terminator codon with an attL sequence included in the primers as described previously^14^. We used the Gateway® system (Thermo Fisher Scientific) for cloning and the amplified attL conjugated genes were cloned into the pEGFP-DEST vector in which the EGFP gene was fused in-frame after recombination as described previously^14^.

### p62 inducer screening

N2a stably transfected with p62PR-EGFP cells were seeded into 96-well plates in 150 µL of culture medium (DMEM/10% FBS) at density of 1 × 10^4^ cells per well. The Sigma LOPAC^®^1280 library (Sigma-Aldrich, St. Louis, MO, USA), containing 1280 pharmacologically active compounds with known mechanisms, was used for screening. Compounds were diluted from the original plates (stock concentration was 10 mM) with DMEM to a final concentration of 1 mM and the diluted compounds were added to the cell culture at a final concentration of 10 µM. Six independent dimethyl sulfoxide (DMSO)-negative controls and 2 µM MG132 (Calbiochem, San Diego, CA, USA)-positive controls were prepared in each assay plate for analysis. For the first screening, cells were treated with each compound for 24 h followed by fixation with 4% paraformaldehyde in phosphate-buffered saline (PBS) and subjected to image-based cellular fluorescence measurement. Each compound was tested in three replicates. The fluorescence cell images were automatically acquired in three different fields per well and analyzed with ArrayScan™ HCS Reader (Cellomics, Thermo Fisher Scientific) according to the manufacturer’s instructions. For data analysis, the mean values of EGFP intensity per cell in each well were divided by the mean EGFP intensity of DMSO controls and fold-induction was calculated. For the second screening, 78 compounds that significantly induced or reduced EGFP fluorescence by more than 1.3-fold were selected. N2a RFPu cells were treated with these selected compounds for 24 h. RFP fluorescence in the fixed cells was measured by ArrayScan™. Only OLDA showed cytoplasmic accumulation of RFP fluorescence among the 78 compounds.

### Antibodies and reagents

Anti-phospho-p62 (S403) clone 4F6 rat monoclonal antibodies were previously described^13^. We prepared the antibody from culture media of 4F6 hybridoma and the antibodies were concentrated by 10-fold from the media using 40% ammonium sulfate (Wako) followed by passing through a desalting column (PD-10; GE Healthcare, Little Chalfont, UK). We used home-made anti-phospho-p62 (S403) antibodies that were 5-fold more concentrated than the commercially available antibodies (Millipore, Billerica, MA, USA, MABC186 and MBL International, Woburn, MA, USA, D343-3). Anti-p62 (polyclonal PM045 for western blotting, and polyclonal C-terminal p62 PM066 for immunofluorescence, MBL International), anti-multiubiquitin (FK2, MBL International), anti-Lamp-2 (ABL-93, Southern Biotech, Birmingham, AL, USA), anti-γ-tubulin (GTU-88, Sigma), anti-ubiquitin (monomer; polyclonal, DAKO, Glostrup, Denmark), anti-HDAC6 (D-11, Santa Cruz Biotechnology, Dallas, TX, USA), and anti-beta-actin (Wako) were purchased from the indicated vendors.

*N*-Oleoyl-dopamine, *N*-Arachidonyl-dopamine, and anandamide were purchased from Cayman Chemical (Ann Arbor, MI, USA) and capsaicin was purchased from Sigma-Aldrich. MG132 and nocodazole were obtained from Calbiochem. BX795 was purchased from Axon Medchem (Reston, VA, USA).

### Immunofluorescence microscopy

For co-localization studies, cells were grown on coverslips coated with poly-L-lysine (Sigma) in 24-well plates. Drug-treated N2a-derived cell lines were fixed in neutralized formaldehyde (Wako) and blocked with 1% FBS and 0.1% Triton X-100 in PBS with 200 mM imidazole, 100 mM NaF, and protease inhibitor cocktail (Roche, Basel, Switzerland). Fixed cells with 4% formaldehyde were incubated with appropriate primary antibodies in blocking buffer, and then with AlexaFluor 488-, 568-, or 647-conjugated anti-rabbit, rat, or mouse IgG (Life Technologies, Carlsbad, CA, USA) after washing with PBS + 0.1% Triton X-100 and mounted in ProLong® Diamond antifade mountant (Thermo Fisher Scientific). Confocal microscopy was performed using a Zeiss LSM710 inverted confocal microscope (Oberkochen, Germany) equipped with a 100x oil lens with 2x magnification. A whole-cell Z stack (each slice = 0.33 μm) was acquired, and a maximum projection was created to visualize all fluorophores in a cell. For co-localization analysis, single confocal layer images were used to generate the magnified images to exclude signals in different confocal layers. Structured illumination microscopy (super-resolution microscopy) was performed using a Zeiss ELYRA super-resolution microscope equipped with a 100x oil lens (NA1.46) (Carl Zeiss). A whole-cell Z stack (each slice = 0.11 μm) was acquired with three rotations and analyzed for reconstruction of super-resolution images. A maximum projection was created to visualize all fluorophores in a cell. All images were processed using Zen (Carl Zeiss) and ImageJ 64 software (NIH, Bethesda, MD, USA).

### Quantitative PCR analysis

Quantitative PCR analysis was performed using a LightCycler 480 and FastStart Universal SYBR Green Master (Roche Molecular Systems) according to the manufacturer’s protocol. Primer sets were designed using the Roche Universal Probe Library Assay Design Center program (Roche Molecular Systems) and at least 2 different primer sets were designed for each gene. We used the following accession numbers for primer design: mp62/SQSTM1/A170: NM_011018, mNBR1: NM_008676, mOPTN: NM_181848, mNDP52: NM_001100177, mTAX1BP1: NM_025816, mNqo1: NM_008706, mLC3B: NM_026160, mGapdh: NM_008084. Sequence information was also used to clone each gene. Gapdh was used for normalization. For absolute amount quantification of autophagy receptors, we used GFP-fused autophagy receptor plasmids, in which the open reading frame sequence of each mouse autophagy receptor was cloned into the pEGFP-C1 plasmid, and then generated a standard curve to quantify the copy number of each receptor. The copy number of plasmid DNA was further normalized to the GFP gene. The PCR amplification standard curve of each autophagy receptor was generated with serially diluted plasmids, and the absolute amount of mRNA in the same mRNA pool was calculated to compare the mRNA amount with different primer sets. At least two different primer sets were used for quantification. For RNA preparation, we used TRIzol® reagent (Thermo Fisher Scientific) and Direct-zol™ RNA Kits (Zymo Research, Irvine, CA, USA) for purification.

### Proteasome degradation assay

*In vitro* proteasome degradation assays were described previously^48,49^. Briefly, 2 nM 26S yeast proteasome and 125 nM Suc-LLVY-AMC in degradation buffer (50 mM Tris-HCl (pH 7.5), 5% (v/v) glycerol, 5 mM MgCl_2_, 1 mM DTT, 1 mM ATP, and 1% DMSO) were incubated at 30 °C and emission fluorescence was measured at 460 nm following excitation at 360 nm for 30 min in 5-min intervals. A total of 9–27 measurements were carried out under each condition.

## Electronic supplementary material

Supplementary Figures
