# Supplementary material for: N-Acyldopamine induces aggresome formation without proteasome inhibition and enhances protein aggregation via p62/SQSTM1 expression
Source: Sci Rep. 2018 Jun 25;8:9585. doi: 10.1038/s41598-018-27872-6 (PMC6018635; doi:10.1038/s41598-018-27872-6)

Supplemental information

N-Acyldopamine induces aggresome formation without proteasome inhibition and enhances protein aggregation via p62/SQSTM1 expression

Gen Matsumoto, Tomonao Inobe, Takanori Amano, Kiyohito Murai, Nobuyuki Nukina, and Nozomu Mori

Supplementary Figures

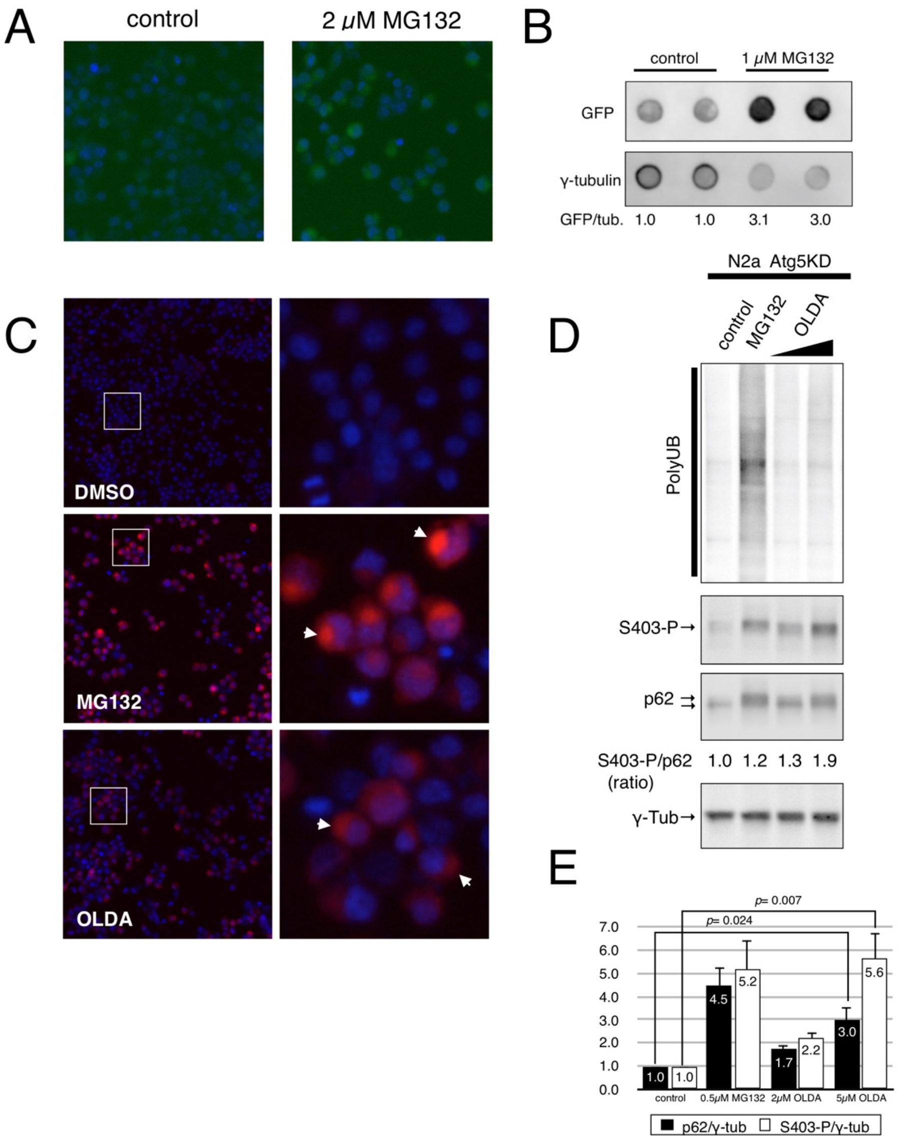

Figure S1: Screening for p62 promoter activators

A. A representative image of GFP accumulation in N2a p62PR-GFP cells with or without 2 μM MG132 or DMSO treatment for 24 hours as positive or negative control for the compound screening, respectively. Images were acquired automatically, then intensity of GFP fluorescence was measured in each cell on the image. **B.** Dot blot analysis for cells

described in B. **C.** Representative images of RFPu cells treated with 1 μM MG132 or 10 μM OLDA for 24 hours upon the screening, as indicated. These images were automatically acquired by Arrayscan image analyzer. Arrows indicate the aggresome-like structure. **D.** N2a Atg5KD cell lines, in which the microRNA derived RNAi constructs against Atg5 were stably transfected in N2a cells, were treated with 1 μM MG132, 2μM OLDA, or 5 μM OLDA for 24 hours and whole cell lysates were subjected to the immunoblotting using anti-ubiquitin (polyUB), anti-phospho-p62 (S403), anti-p62, or anti-gamma-tubulin. The Ratio between S403-phosphoryalted p62 and total p62 were shown. **E.** Relative amount of S403-phospho-p62 and total p62 shown in D were measured. Error bars represent SEM (n>4) and p values (Student's t-test) was shown.

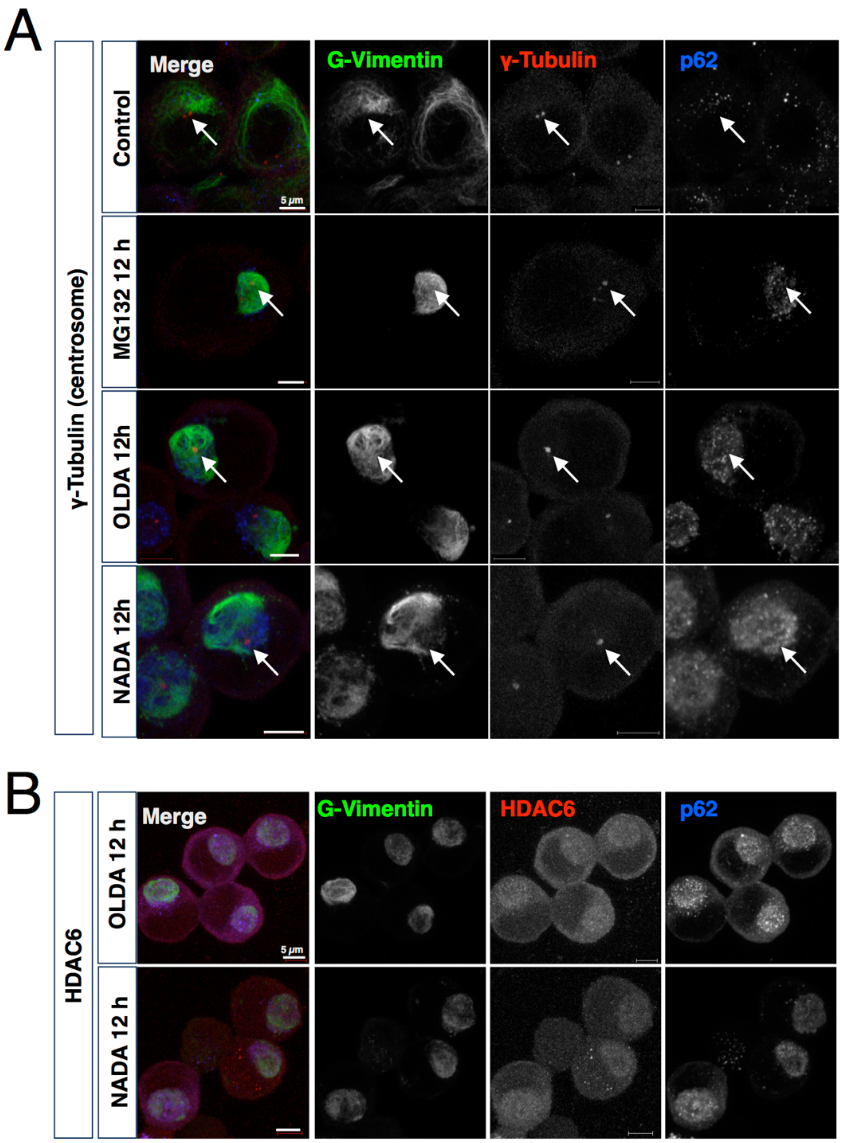

Figure S2: Aggresome markers are detected in the AcylDA-induced aggresomes. N2a cells stably expressing GFP-Vimentin cell lines were treated with 1 µM MG132, 5µM OLDA, or 5 µM NADA for 12 hours as indicated. G-Vimentin (green), gannma-tubulin (MTOC, red) in **A**, HDAC6 (red) in **B**, p62 (blue), and S403-phosphorylated p62 (S403-P; blue) were visualized by confocal microscopy as indicated. Arrows indicate the mitotic organizing center (MTOC, centrosomes). Scale bars represent 5 µm

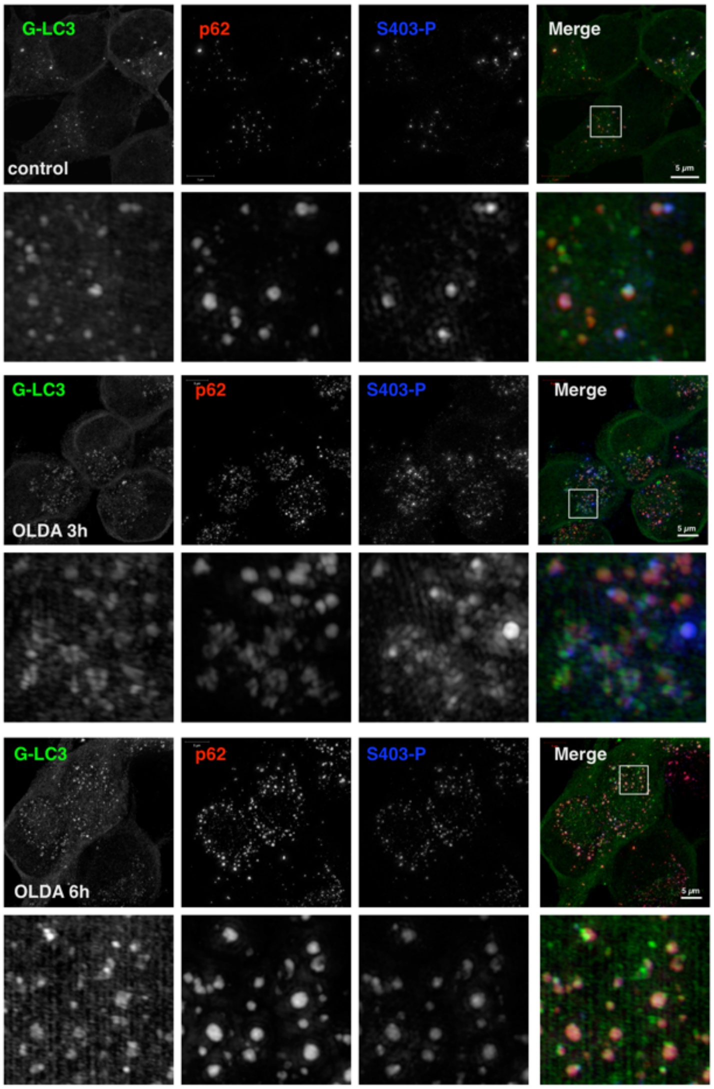

Figure S3: Autophagosomal engulfment of S403-phospho-p62 by super-resolution structured illumination microscopy.

N2a cells stably expressing GFP fused mouse LC3B (G-LC3) were treated with 5μM OLDA for 3 or 6 hours as indicated. G-LC3 (green), total p62 (red), and S403-phosphorylated p62 (S403-P; blue) were visualized by the super resolution structured illumination microscopy as indicated. Magnified images in square box (5 μm each side) were shown. Scale bar represents 5 μm in wide images.

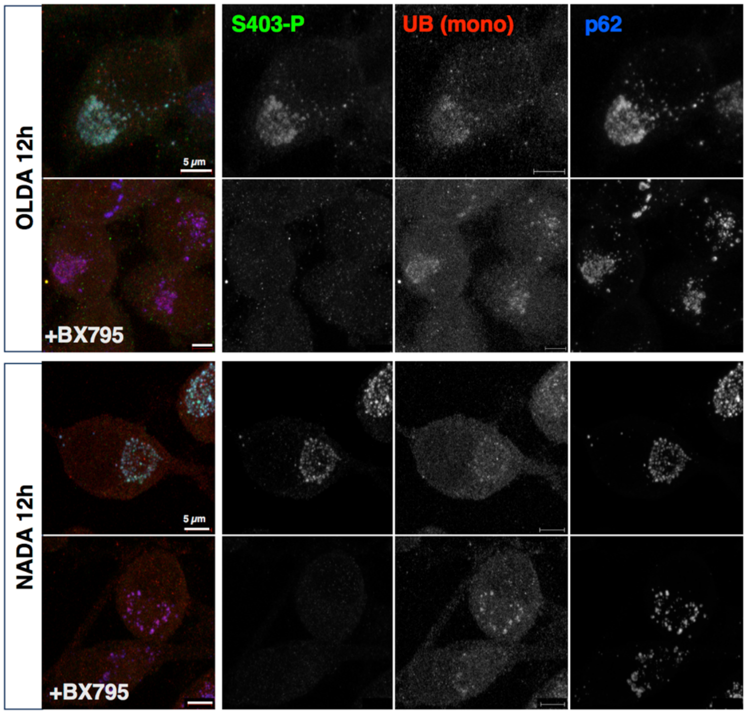

Figure S4: S403-phosphorylation of p62 is dispensable for the AcylDA-mediated aggresome formation and the inhibition of TBK1 prevent the S403-phosphorylation of p62.

N2a cells were treated with or without 5µM OLDA, 5µM NADA, or 1 µM BX795 for 12 hours as indicated. S403-phosphorylated p62 (S403-P; green), ubiquitin monomer (UB mono, red), and total p62 (blue) were visualized by confocal microscopy as indicated. Scale bar represents 5 µm in wide images.

Figure1C:

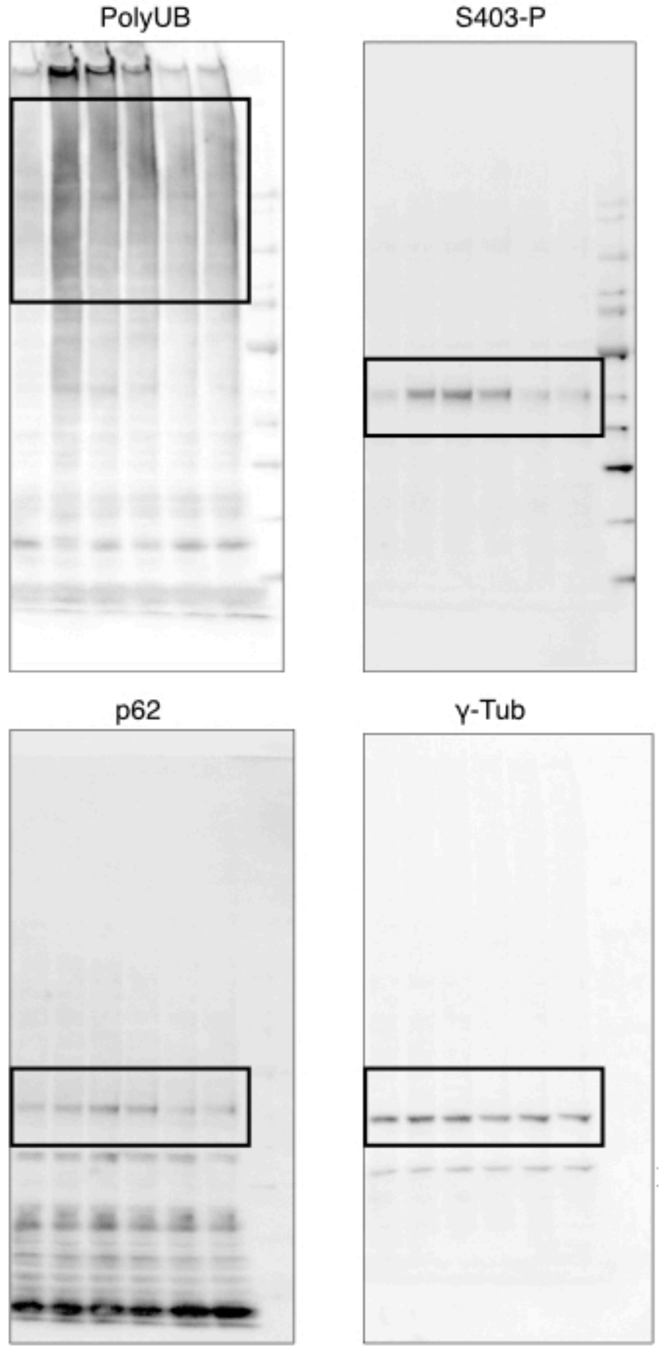

Figure1D

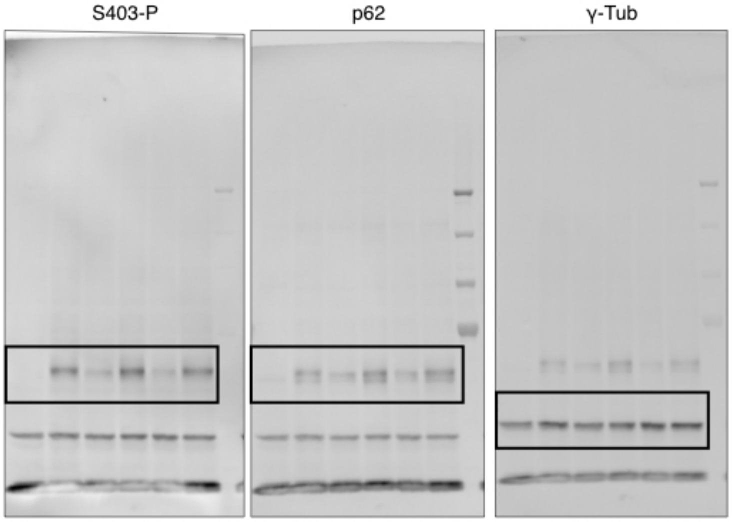

Figure6C

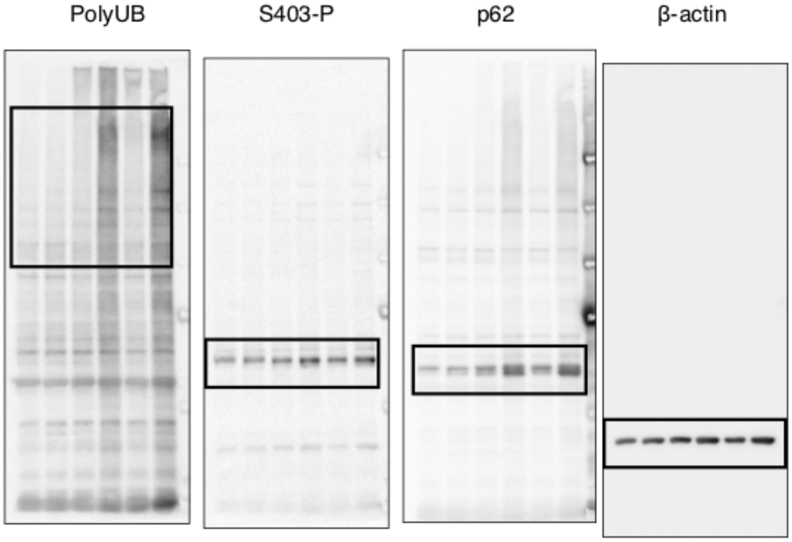

Supplement: Supplementary file 1 — Supplementary Figures [file 41598_2018_27872_MOESM1_ESM.pdf]
